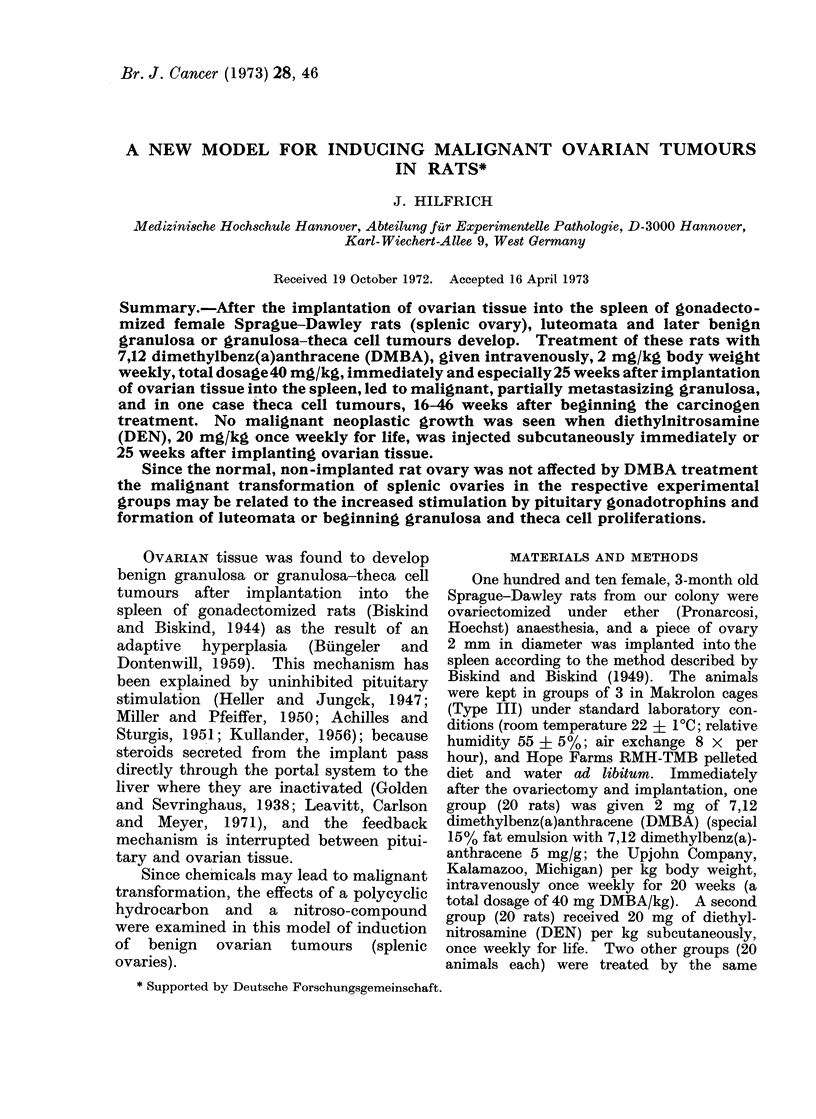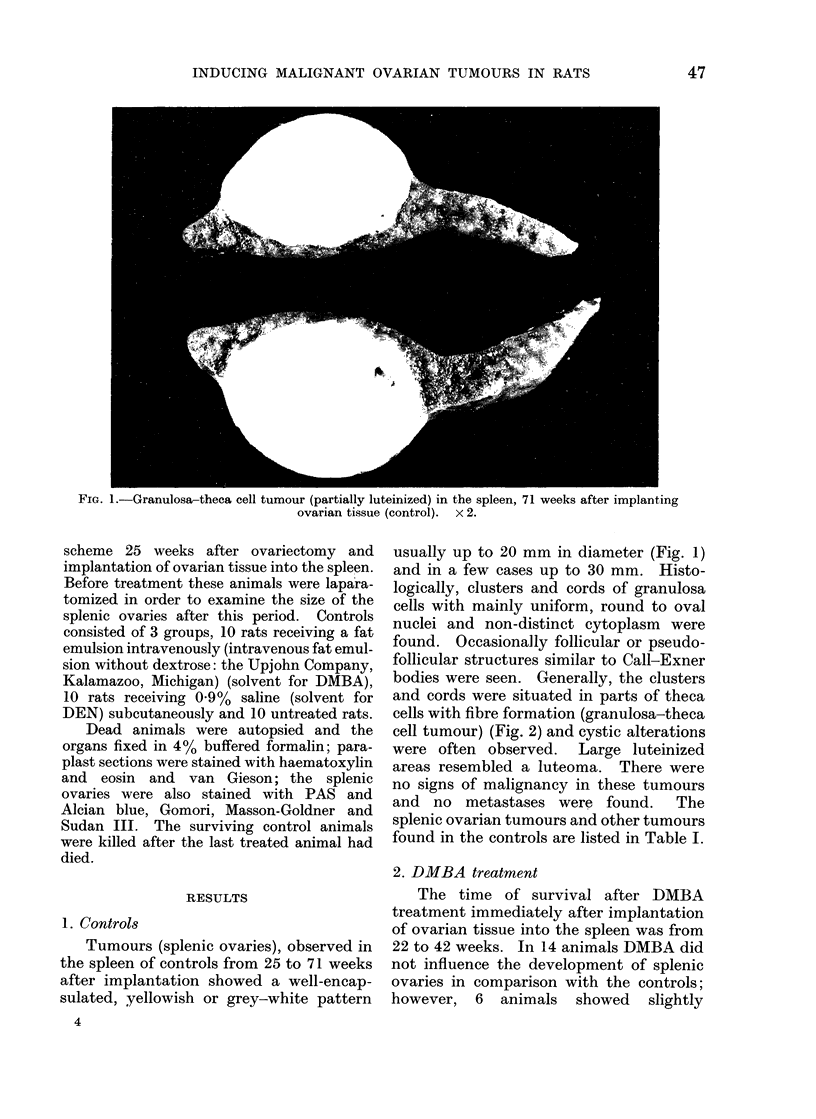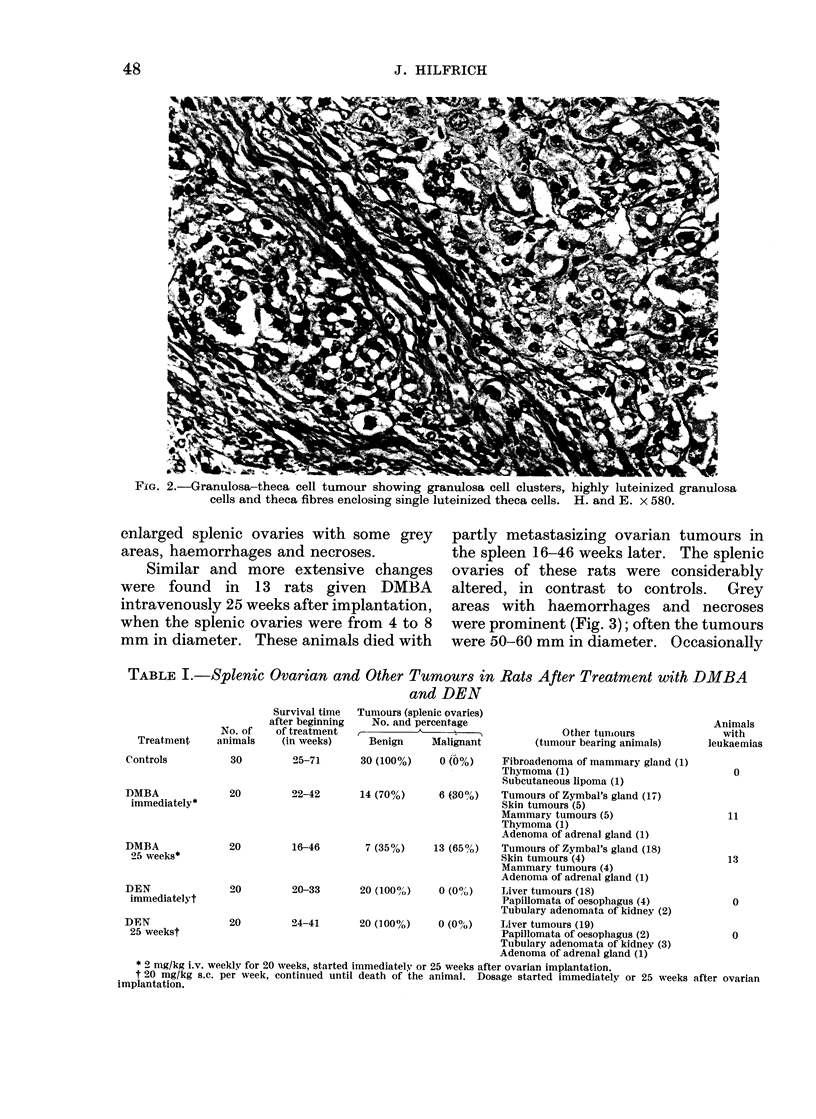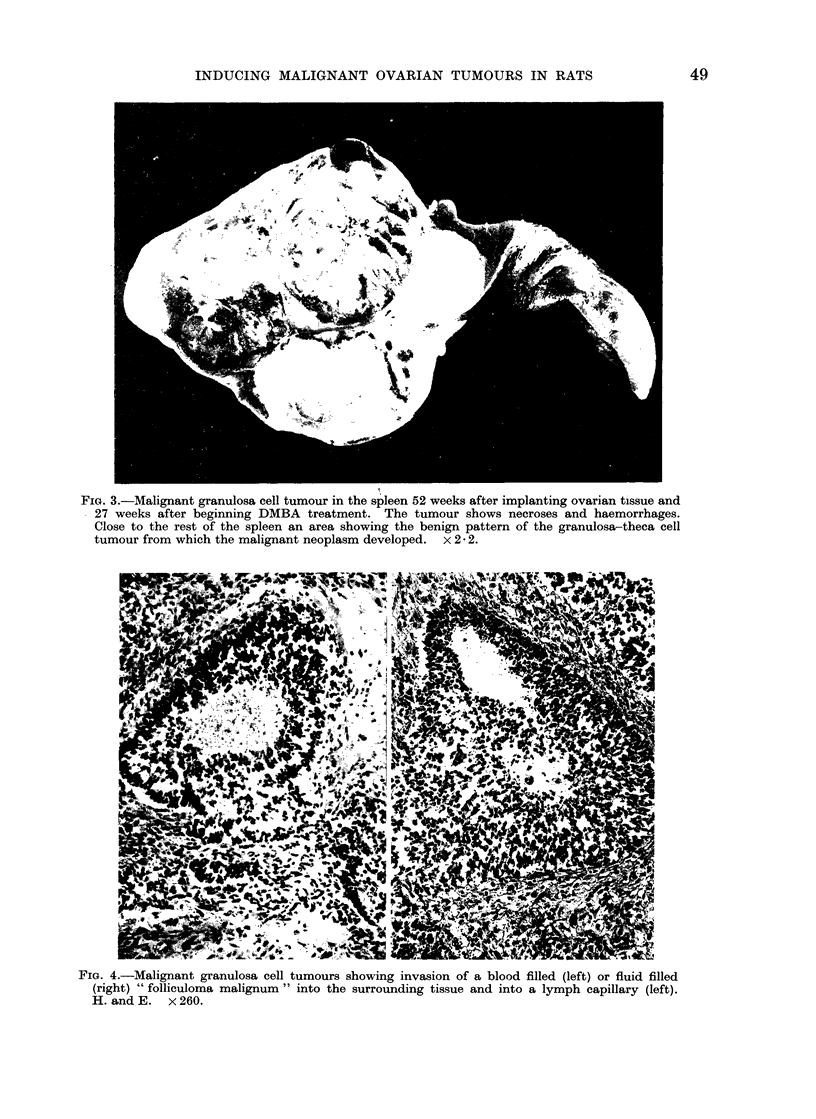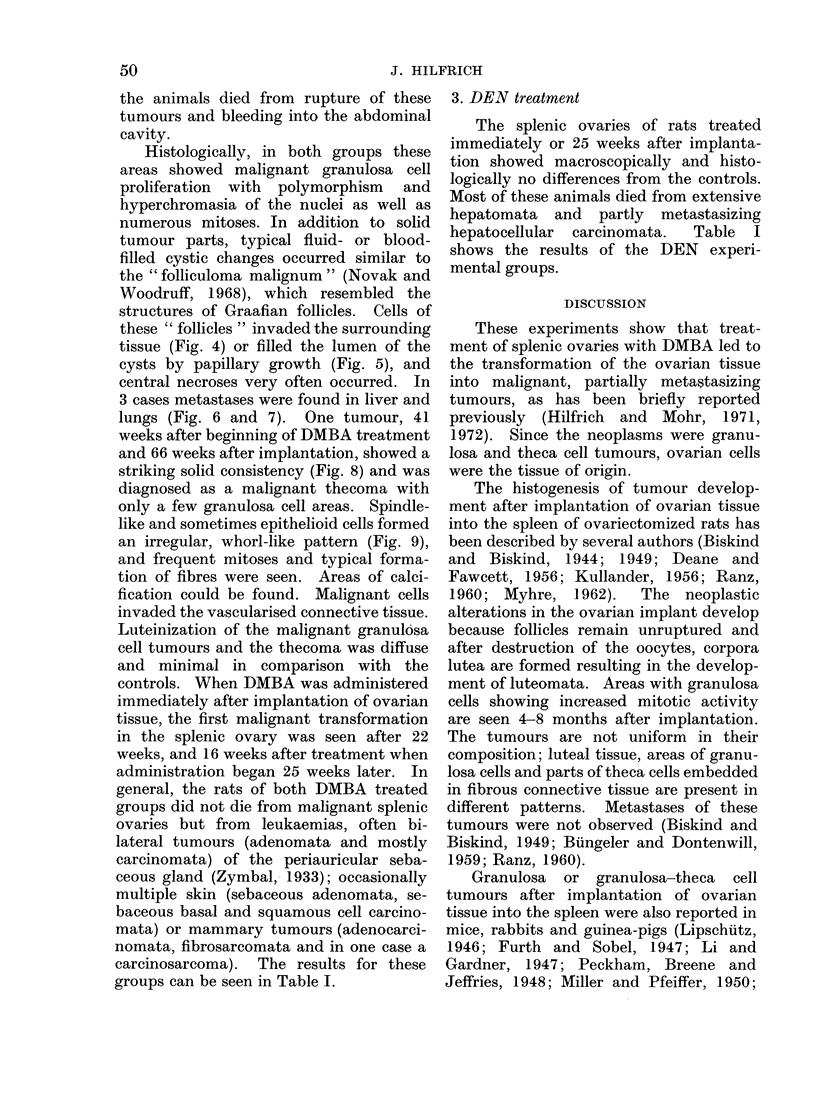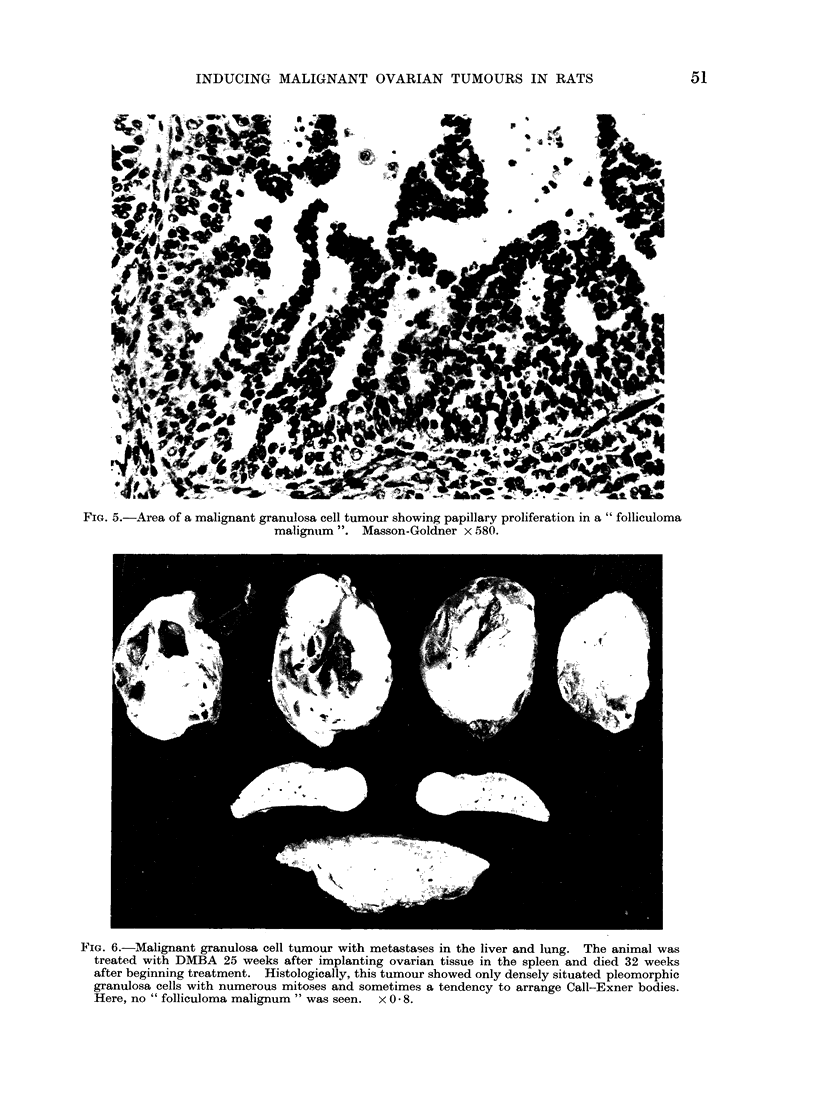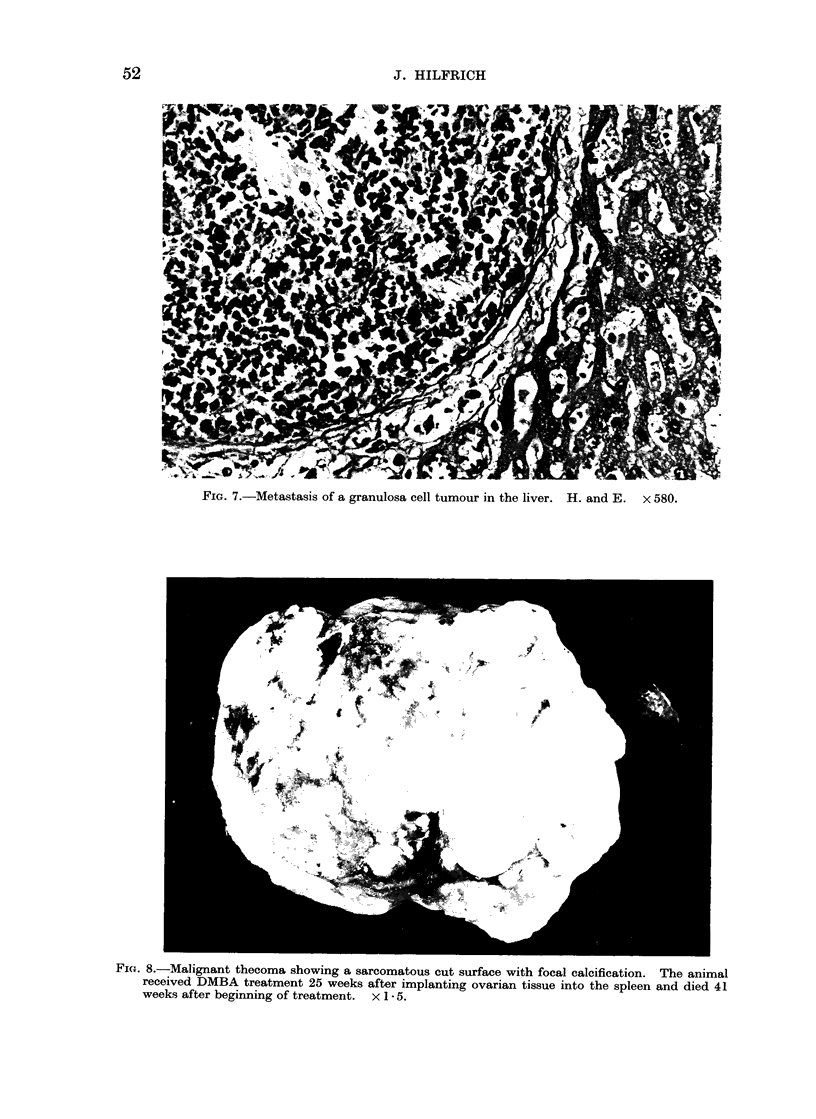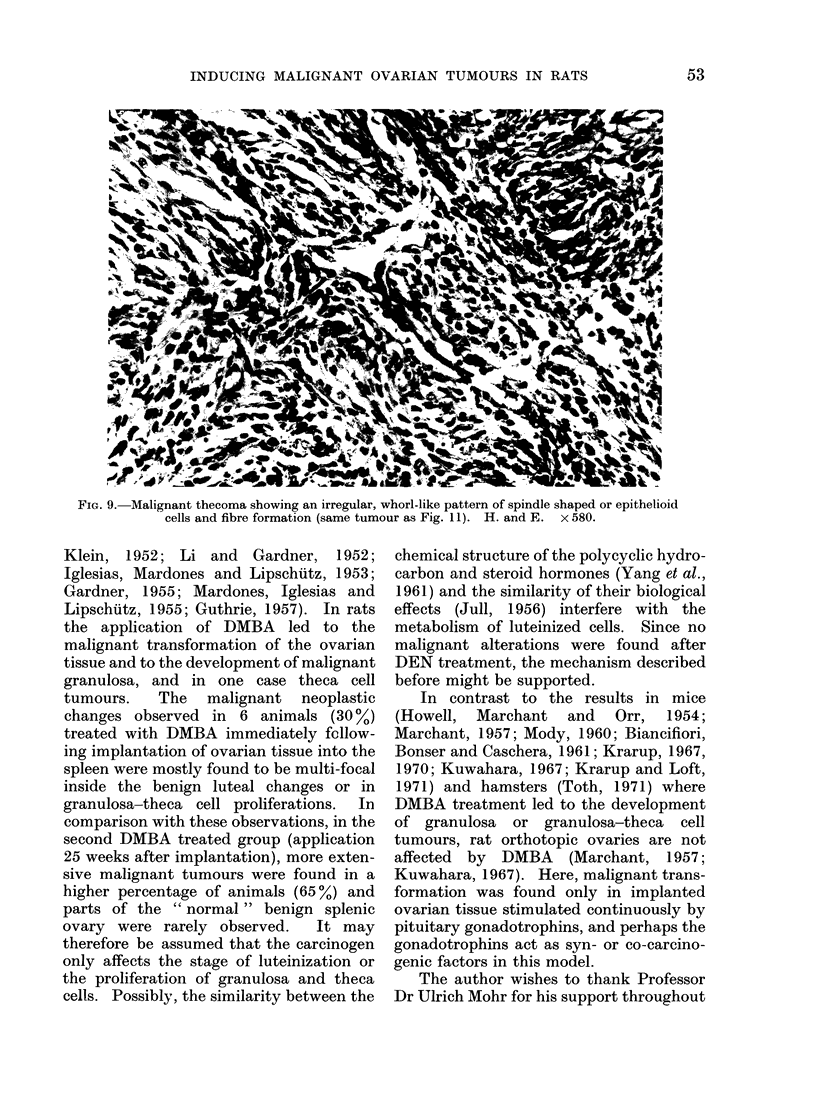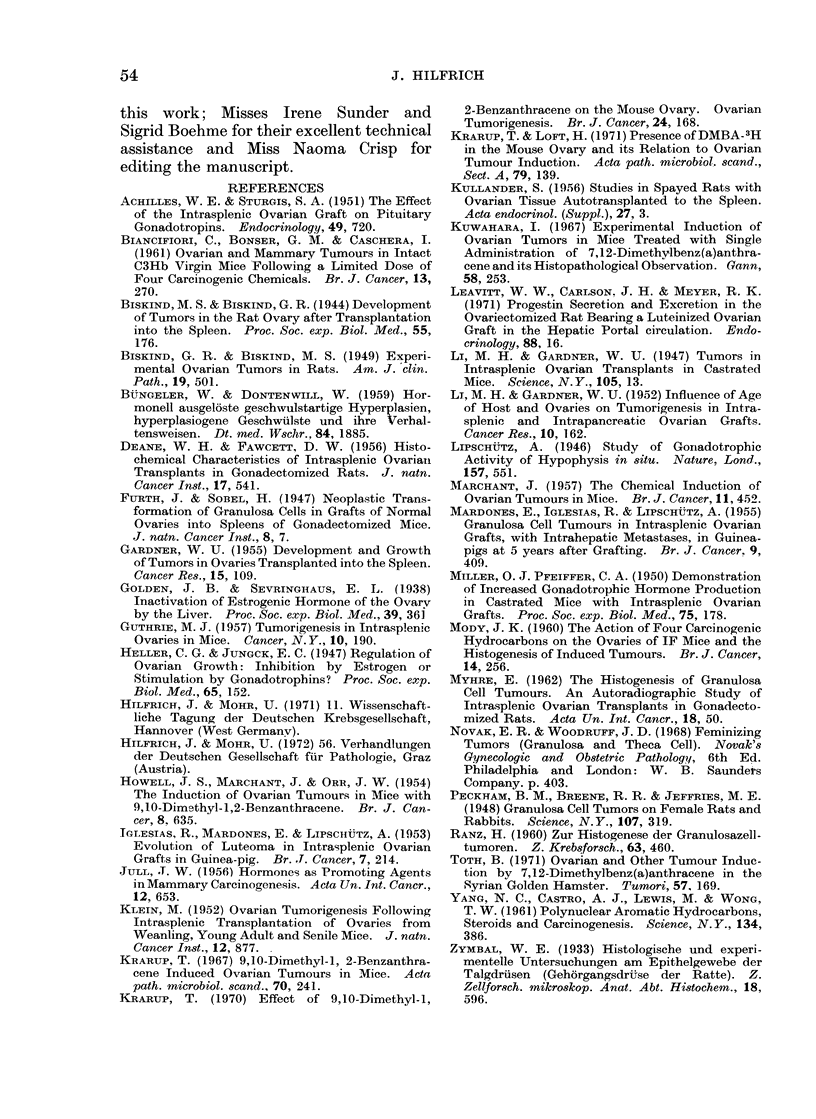# A New Model for Inducing Malignant Ovarian Tumours in Rats*

**DOI:** 10.1038/bjc.1973.69

**Published:** 1973-07

**Authors:** J. Hilfrich

## Abstract

**Images:**


*Br. J. Cancer* (1973) **28**, 46

### A NEW MODEL FOR INDUCING MALIGNANT OVARIAN TUMOURS IN RATS*

J. HILFRICH

*Medizinische Hochschule Hannover, Abteilung für Experimentelle Pathologie, D-3000 Hannover, Karl-Wiechert-Allee 9, West Germany*

Received 19 October 1972. Accepted 16 April 1973

**Summary.—After the implantation of ovarian tissue into the spleen of gonadectomized female Sprague–Dawley rats (splenic ovary), luteomata and later benign granulosa or granulosa–theca cell tumours develop. Treatment of these rats with 7,12 dimethylbenz(a)anthracene (DMBA), given intravenously, 2 mg/kg body weight weekly, total dosage 40 mg/kg, immediately and especially 25 weeks after implantation of ovarian tissue into the spleen, led to malignant, partially metastasizing granulosa, and in one case theca cell tumours, 16–46 weeks after beginning the carcinogen treatment. No malignant neoplastic growth was seen when diethylnitrosamine (DEN), 20 mg/kg once weekly for life, was injected subcutaneously immediately or 25 weeks after implanting ovarian tissue.**

**Since the normal, non-implanted rat ovary was not affected by DMBA treatment the malignant transformation of splenic ovaries in the respective experimental groups may be related to the increased stimulation by pituitary gonadotrophins and formation of luteomata or beginning granulosa and theca cell proliferations.**

OVARIAN tissue was found to develop benign granulosa or granulosa–theca cell tumours after implantation into the spleen of gonadectomized rats (Biskind and Biskind, 1944) as the result of an adaptive hyperplasia (Büngeler and Dontenwill, 1959). This mechanism has been explained by uninhibited pituitary stimulation (Heller and Jungck, 1947; Miller and Pfeiffer, 1950; Achilles and Sturgis, 1951; Kullander, 1956); because steroids secreted from the implant pass directly through the portal system to the liver where they are inactivated (Golden and Sevringhaus, 1938; Leavitt, Carlson and Meyer, 1971), and the feedback mechanism is interrupted between pituitary and ovarian tissue.

Since chemicals may lead to malignant transformation, the effects of a polycyclic hydrocarbon and a nitroso-compound were examined in this model of induction of benign ovarian tumours (splenic ovaries).

### MATERIALS AND METHODS

One hundred and ten female, 3-month old Sprague–Dawley rats from our colony were ovariectomized under ether (Pronarcosi, Hoechst) anaesthesia, and a piece of ovary 2 mm in diameter was implanted into the spleen according to the method described by Biskind and Biskind (1949). The animals were kept in groups of 3 in Makrolon cages (Type III) under standard laboratory conditions (room temperature 22 ± 1°C; relative humidity 55 ± 5%; air exchange 8 × per hour), and Hope Farms RMH-TMB pelleted diet and water *ad libitum*. Immediately after the ovariectomy and implantation, one group (20 rats) was given 2 mg of 7,12 dimethylbenz(a)anthracene (DMBA) (special 15% fat emulsion with 7,12 dimethylbenz(a)-anthracene 5 mg/g; the Upjohn Company, Kalamazoo, Michigan) per kg body weight, intravenously once weekly for 20 weeks (a total dosage of 40 mg DMBA/kg). A second group (20 rats) received 20 mg of diethylnitrosamine (DEN) per kg subcutaneously, once weekly for life. Two other groups (20 animals each) were treated by the same

* Supported by Deutsche Forschungsgemeinschaft.

FIG. 1.—Granulosa–theca cell tumour (partially luteinized) in the spleen, 71 weeks after implanting ovarian tissue (control). × 2.

scheme 25 weeks after ovariectomy and implantation of ovarian tissue into the spleen. Before treatment these animals were laparatomized in order to examine the size of the splenic ovaries after this period. Controls consisted of 3 groups, 10 rats receiving a fat emulsion intravenously (intravenous fat emulsion without dextrose: the Upjohn Company, Kalamazoo, Michigan) (solvent for DMBA), 10 rats receiving 0·9% saline (solvent for DEN) subcutaneously and 10 untreated rats.

Dead animals were autopsied and the organs fixed in 4% buffered formalin; paraplast sections were stained with haematoxylin and eosin and van Gieson; the splenic ovaries were also stained with PAS and Alcian blue, Gomori, Masson-Goldner and Sudan III. The surviving control animals were killed after the last treated animal had died.

### RESULTS

### 1. *Controls*

Tumours (splenic ovaries), observed in the spleen of controls from 25 to 71 weeks after implantation showed a well-encapsulated, yellowish or grey–white pattern usually up to 20 mm in diameter (Fig. 1) and in a few cases up to 30 mm. Histologically, clusters and cords of granulosa cells with mainly uniform, round to oval nuclei and non-distinct cytoplasm were found. Occasionally follicular or pseudofollicular structures similar to Call–Exner bodies were seen. Generally, the clusters and cords were situated in parts of theca cells with fibre formation (granulosa–theca cell tumour) (Fig. 2) and cystic alterations were often observed. Large luteinized areas resembled a luteoma. There were no signs of malignancy in these tumours and no metastases were found. The splenic ovarian tumours and other tumours found in the controls are listed in Table I.

### 2. *DMBA treatment*

The time of survival after DMBA treatment immediately after implantation of ovarian tissue into the spleen was from 22 to 42 weeks. In 14 animals DMBA did not influence the development of splenic ovaries in comparison with the controls; however, 6 animals showed slightly

FIG. 2.—Granulosa–theca cell tumour showing granulosa cell clusters, highly luteinized granulosa cells and theca fibres enclosing single luteinized theca cells. H. and E. ×580.

enlarged splenic ovaries with some grey areas, haemorrhages and necroses.

Similar and more extensive changes were found in 13 rats given DMBA intravenously 25 weeks after implantation, when the splenic ovaries were from 4 to 8 mm in diameter. These animals died with partly metastasizing ovarian tumours in the spleen 16–46 weeks later. The splenic ovaries of these rats were considerably altered, in contrast to controls. Grey areas with haemorrhages and necroses were prominent (Fig. 3); often the tumours were 50–60 mm in diameter. Occasionally

TABLE I.—*Splenic Ovarian and Other Tumours in Rats After Treatment with DMBA and DEN*

| Treatment | No. of animals | Survival time after beginning of treatment (in weeks) | Tumours (splenic ovaries) No. and percentage | | Other tumours (tumour bearing animals) | Animals with leukaemias |
|---|---|---|---|---|---|---|
| | | | Benign | Malignant | | |
| Controls | 30 | 25–71 | 30 (100%) | 0 (0%) | Fibroadenoma of mammary gland (1)<br>Thymoma (1)<br>Subcutaneous lipoma (1) | 0 |
| DMBA immediately* | 20 | 22–42 | 14 (70%) | 6 (30%) | Tumours of Zymbal's gland (17)<br>Skin tumours (5)<br>Mammary tumours (5)<br>Thymoma (1)<br>Adenoma of adrenal gland (1) | 11 |
| DMBA 25 weeks* | 20 | 16–46 | 7 (35%) | 13 (65%) | Tumours of Zymbal's gland (18)<br>Skin tumours (4)<br>Mammary tumours (4)<br>Adenoma of adrenal gland (1) | 13 |
| DEN immediately† | 20 | 20–33 | 20 (100%) | 0 (0%) | Liver tumours (18)<br>Papillomata of oesophagus (4)<br>Tubulary adenomata of kidney (2) | 0 |
| DEN 25 weeks† | 20 | 24–41 | 20 (100%) | 0 (0%) | Liver tumours (19)<br>Papillomata of oesophagus (2)<br>Tubulary adenomata of kidney (3)<br>Adenoma of adrenal gland (1) | 0 |

* 2 mg/kg i.v. weekly for 20 weeks, started immediately or 25 weeks after ovarian implantation.

† 20 mg/kg s.c. per week, continued until death of the animal. Dosage started immediately or 25 weeks after ovarian implantation.

FIG. 3.—Malignant granulosa cell tumour in the spleen 52 weeks after implanting ovarian tissue and 27 weeks after beginning DMBA treatment. The tumour shows necroses and haemorrhages. Close to the rest of the spleen an area showing the benign pattern of the granulosa–theca cell tumour from which the malignant neoplasm developed. ×2·2.

FIG. 4.—Malignant granulosa cell tumours showing invasion of a blood filled (left) or fluid filled (right) " folliculoma malignum " into the surrounding tissue and into a lymph capillary (left). H. and E. ×260.

the animals died from rupture of these tumours and bleeding into the abdominal cavity.

Histologically, in both groups these areas showed malignant granulosa cell proliferation with polymorphism and hyperchromasia of the nuclei as well as numerous mitoses. In addition to solid tumour parts, typical fluid- or blood-filled cystic changes occurred similar to the "folliculoma malignum" (Novak and Woodruff, 1968), which resembled the structures of Graafian follicles. Cells of these "follicles" invaded the surrounding tissue (Fig. 4) or filled the lumen of the cysts by papillary growth (Fig. 5), and central necroses very often occurred. In 3 cases metastases were found in liver and lungs (Fig. 6 and 7). One tumour, 41 weeks after beginning of DMBA treatment and 66 weeks after implantation, showed a striking solid consistency (Fig. 8) and was diagnosed as a malignant thecoma with only a few granulosa cell areas. Spindle-like and sometimes epithelioid cells formed an irregular, whorl-like pattern (Fig. 9), and frequent mitoses and typical formation of fibres were seen. Areas of calcification could be found. Malignant cells invaded the vascularised connective tissue. Luteinization of the malignant granulosa cell tumours and the thecoma was diffuse and minimal in comparison with the controls. When DMBA was administered immediately after implantation of ovarian tissue, the first malignant transformation in the splenic ovary was seen after 22 weeks, and 16 weeks after treatment when administration began 25 weeks later. In general, the rats of both DMBA treated groups did not die from malignant splenic ovaries but from leukaemias, often bilateral tumours (adenomata and mostly carcinomata) of the periauricular sebaceous gland (Zymbal, 1933); occasionally multiple skin (sebaceous adenomata, sebaceous basal and squamous cell carcinomata) or mammary tumours (adenocarcinomata, fibrosarcomata and in one case a carcinosarcoma). The results for these groups can be seen in Table I.

3. *DEN treatment*

The splenic ovaries of rats treated immediately or 25 weeks after implantation showed macroscopically and histologically no differences from the controls. Most of these animals died from extensive hepatomata and partly metastasizing hepatocellular carcinomata. Table I shows the results of the DEN experimental groups.

### DISCUSSION

These experiments show that treatment of splenic ovaries with DMBA led to the transformation of the ovarian tissue into malignant, partially metastasizing tumours, as has been briefly reported previously (Hilfrich and Mohr, 1971, 1972). Since the neoplasms were granulosa and theca cell tumours, ovarian cells were the tissue of origin.

The histogenesis of tumour development after implantation of ovarian tissue into the spleen of ovariectomized rats has been described by several authors (Biskind and Biskind, 1944; 1949; Deane and Fawcett, 1956; Kullander, 1956; Ranz, 1960; Myhre, 1962). The neoplastic alterations in the ovarian implant develop because follicles remain unruptured and after destruction of the oocytes, corpora lutea are formed resulting in the development of luteomata. Areas with granulosa cells showing increased mitotic activity are seen 4–8 months after implantation. The tumours are not uniform in their composition; luteal tissue, areas of granulosa cells and parts of theca cells embedded in fibrous connective tissue are present in different patterns. Metastases of these tumours were not observed (Biskind and Biskind, 1949; Büngeler and Dontenwill, 1959; Ranz, 1960).

Granulosa or granulosa–theca cell tumours after implantation of ovarian tissue into the spleen were also reported in mice, rabbits and guinea-pigs (Lipschütz, 1946; Furth and Sobel, 1947; Li and Gardner, 1947; Peckham, Breene and Jeffries, 1948; Miller and Pfeiffer, 1950;

FIG. 5.—Area of a malignant granulosa cell tumour showing papillary proliferation in a " folliculoma malignum ". Masson-Goldner ×580.

FIG. 6.—Malignant granulosa cell tumour with metastases in the liver and lung. The animal was treated with DMBA 25 weeks after implanting ovarian tissue in the spleen and died 32 weeks after beginning treatment. Histologically, this tumour showed only densely situated pleomorphic granulosa cells with numerous mitoses and sometimes a tendency to arrange Call–Exner bodies. Here, no " folliculoma malignum " was seen. ×0·8.

FIG. 7.—Metastasis of a granulosa cell tumour in the liver. H. and E. ×580.

FIG. 8.—Malignant thecoma showing a sarcomatous cut surface with focal calcification. The animal received DMBA treatment 25 weeks after implanting ovarian tissue into the spleen and died 41 weeks after beginning of treatment. ×1·5.

FIG. 9.—Malignant thecoma showing an irregular, whorl-like pattern of spindle shaped or epithelioid cells and fibre formation (same tumour as Fig. 11). H. and E. ×580.

Klein, 1952; Li and Gardner, 1952; Iglesias, Mardones and Lipschütz, 1953; Gardner, 1955; Mardones, Iglesias and Lipschütz, 1955; Guthrie, 1957). In rats the application of DMBA led to the malignant transformation of the ovarian tissue and to the development of malignant granulosa, and in one case theca cell tumours. The malignant neoplastic changes observed in 6 animals (30%) treated with DMBA immediately following implantation of ovarian tissue into the spleen were mostly found to be multi-focal inside the benign luteal changes or in granulosa–theca cell proliferations. In comparison with these observations, in the second DMBA treated group (application 25 weeks after implantation), more extensive malignant tumours were found in a higher percentage of animals (65%) and parts of the "normal" benign splenic ovary were rarely observed. It may therefore be assumed that the carcinogen only affects the stage of luteinization or the proliferation of granulosa and theca cells. Possibly, the similarity between the chemical structure of the polycyclic hydrocarbon and steroid hormones (Yang *et al.*, 1961) and the similarity of their biological effects (Jull, 1956) interfere with the metabolism of luteinized cells. Since no malignant alterations were found after DEN treatment, the mechanism described before might be supported.

In contrast to the results in mice (Howell, Marchant and Orr, 1954; Marchant, 1957; Mody, 1960; Biancifiori, Bonser and Caschera, 1961; Krarup, 1967, 1970; Kuwahara, 1967; Krarup and Loft, 1971) and hamsters (Toth, 1971) where DMBA treatment led to the development of granulosa or granulosa–theca cell tumours, rat orthotopic ovaries are not affected by DMBA (Marchant, 1957; Kuwahara, 1967). Here, malignant transformation was found only in implanted ovarian tissue stimulated continuously by pituitary gonadotrophins, and perhaps the gonadotrophins act as syn- or co-carcinogenic factors in this model.

The author wishes to thank Professor Dr Ulrich Mohr for his support throughout this work; Misses Irene Sunder and Sigrid Boehme for their excellent technical assistance and Miss Naoma Crisp for editing the manuscript.

### REFERENCES

ACHILLES, W. E. & STURGIS, S. A. (1951) The Effect of the Intrasplenic Ovarian Graft on Pituitary Gonadotropins. *Endocrinology*, **49**, 720.

BIANCIFIORI, C., BONSER, G. M. & CASCHERA, I. (1961) Ovarian and Mammary Tumours in Intact C3Hb Virgin Mice Following a Limited Dose of Four Carcinogenic Chemicals. *Br. J. Cancer*, **13**, 270.

BISKIND, M. S. & BISKIND, G. R. (1944) Development of Tumors in the Rat Ovary after Transplantation into the Spleen. *Proc. Soc. exp. Biol. Med.*, **55**, 176.

BISKIND, G. R. & BISKIND, M. S. (1949) Experimental Ovarian Tumors in Rats. *Am. J. clin. Path.*, **19**, 501.

BÜNGELER, W. & DONTENWILL, W. (1959) Hormonell ausgelöste geschwulstartige Hyperplasien, hyperplasiogene Geschwülste und ihre Verhaltensweisen. *Dt. med. Wschr.*, **84**, 1885.

DEANE, W. H. & FAWCETT, D. W. (1956) Histochemical Characteristics of Intrasplenic Ovarian Transplants in Gonadectomized Rats. *J. natn. Cancer Inst.*, **17**, 541.

FURTH, J. & SOBEL, H. (1947) Neoplastic Transformation of Granulosa Cells in Grafts of Normal Ovaries into Spleens of Gonadectomized Mice. *J. natn. Cancer Inst.*, **8**, 7.

GARDNER, W. U. (1955) Development and Growth of Tumors in Ovaries Transplanted into the Spleen. *Cancer Res.*, **15**, 109.

GOLDEN, J. B. & SEVRINGHAUS, E. L. (1938) Inactivation of Estrogenic Hormone of the Ovary by the Liver. *Proc. Soc. exp. Biol. Med.*, **39**, 361

GUTHRIE, M. J. (1957) Tumorigenesis in Intrasplenic Ovaries in Mice. *Cancer, N.Y.*, **10**, 190.

HELLER, C. G. & JUNGCK, E. C. (1947) Regulation of Ovarian Growth: Inhibition by Estrogen or Stimulation by Gonadotrophins? *Proc. Soc. exp. Biol. Med.*, **65**, 152.

HILFRICH, J. & MOHR, U. (1971) 11. Wissenschaftliche Tagung der Deutschen Krebsgesellschaft, Hannover (West Germany).

HILFRICH, J. & MOHR, U. (1972) 56. Verhandlungen der Deutschen Gesellschaft für Pathologie, Graz (Austria).

HOWELL, J. S., MARCHANT, J. & ORR, J. W. (1954) The Induction of Ovarian Tumours in Mice with 9,10-Dimethyl-1,2-Benzanthracene. *Br. J. Cancer*, **8**, 635.

IGLESIAS, R., MARDONES, E. & LIPSCHÜTZ, A. (1953) Evolution of Luteoma in Intrasplenic Ovarian Grafts in Guinea-pig. *Br. J. Cancer*, **7**, 214.

JULL, J. W. (1956) Hormones as Promoting Agents in Mammary Carcinogenesis. *Acta Un. Int. Cancr.*, **12**, 653.

KLEIN, M. (1952) Ovarian Tumorigenesis Following Intrasplenic Transplantation of Ovaries from Weanling, Young Adult and Senile Mice. *J. natn. Cancer Inst.*, **12**, 877.

KRARUP, T. (1967) 9,10-Dimethyl-1, 2-Benzanthracene Induced Ovarian Tumours in Mice. *Acta path. microbiol. scand.*, **70**, 241.

KRARUP, T. (1970) Effect of 9,10-Dimethyl-1, 2-Benzanthracene on the Mouse Ovary. Ovarian Tumorigenesis. *Br. J. Cancer*, **24**, 168.

KRARUP, T. & LOFT, H. (1971) Presence of DMBA-$^3$H in the Mouse Ovary and its Relation to Ovarian Tumour Induction. *Acta path. microbiol. scand., Sect. A*, **79**, 139.

KULLANDER, S. (1956) Studies in Spayed Rats with Ovarian Tissue Autotransplanted to the Spleen. *Acta endocrinol. (Suppl.)*, **27**, 3.

KUWAHARA, I. (1967) Experimental Induction of Ovarian Tumors in Mice Treated with Single Administration of 7,12-Dimethylbenz(a)anthracene and its Histopathological Observation. *Gann*, **58**, 253.

LEAVITT, W. W., CARLSON, J. H. & MEYER, R. K. (1971) Progestin Secretion and Excretion in the Ovariectomized Rat Bearing a Luteinized Ovarian Graft in the Hepatic Portal circulation. *Endocrinology*, **88**, 16.

LI, M. H. & GARDNER, W. U. (1947) Tumors in Intrasplenic Ovarian Transplants in Castrated Mice. *Science, N.Y.*, **105**, 13.

LI, M. H. & GARDNER, W. U. (1952) Influence of Age of Host and Ovaries on Tumorigenesis in Intrasplenic and Intrapancreatic Ovarian Grafts. *Cancer Res.*, **10**, 162.

LIPSCHÜTZ, A. (1946) Study of Gonadotrophic Activity of Hypophysis *in situ*. *Nature, Lond.*, **157**, 551.

MARCHANT, J. (1957) The Chemical Induction of Ovarian Tumours in Mice. *Br. J. Cancer*, **11**, 452.

MARDONES, E., IGLESIAS, R. & LIPSCHÜTZ, A. (1955) Granulosa Cell Tumours in Intrasplenic Ovarian Grafts, with Intrahepatic Metastases, in Guinea-pigs at 5 years after Grafting. *Br. J. Cancer*, **9**, 409.

MILLER, O. J. PFEIFFER, C. A. (1950) Demonstration of Increased Gonadotrophic Hormone Production in Castrated Mice with Intrasplenic Ovarian Grafts. *Proc. Soc. exp. Biol. Med.*, **75**, 178.

MODY, J. K. (1960) The Action of Four Carcinogenic Hydrocarbons on the Ovaries of IF Mice and the Histogenesis of Induced Tumours. *Br. J. Cancer*, **14**, 256.

MYHRE, E. (1962) The Histogenesis of Granulosa Cell Tumours. An Autoradiographic Study of Intrasplenic Ovarian Transplants in Gonadectomized Rats. *Acta Un. Int. Cancr.*, **18**, 50.

NOVAK, E. R. & WOODRUFF, J. D. (1968) Feminizing Tumors (Granulosa and Theca Cell). *Novak's Gynecologic and Obstetric Pathology*, 6th Ed. Philadelphia and London: W. B. Saunders Company. p. 403.

PECKHAM, B. M., BREENE, R. R. & JEFFRIES, M. E. (1948) Granulosa Cell Tumors on Female Rats and Rabbits. *Science, N.Y.*, **107**, 319.

RANZ, H. (1960) Zur Histogenese der Granulosazelltumoren. *Z. Krebsforsch.*, **63**, 460.

TOTH, B. (1971) Ovarian and Other Tumour Induction by 7,12-Dimethylbenz(a)anthracene in the Syrian Golden Hamster. *Tumori*, **57**, 169.

YANG, N. C., CASTRO, A. J., LEWIS, M. & WONG, T. W. (1961) Polynuclear Aromatic Hydrocarbons, Steroids and Carcinogenesis. *Science, N.Y.*, **134**, 386.

ZYMBAL, W. E. (1933) Histologische und experimentelle Untersuchungen am Epithelgewebe der Talgdrüsen (Gehörgangsdrüse der Ratte). *Z. Zellforsch. mikroskop. Anat. Abt. Histochem.*, **18**, 596.